# Monitoring the efficacy of artemether-lumefantrine for the treatment of uncomplicated malaria in Malawian children

**DOI:** 10.1186/s12936-015-0701-8

**Published:** 2015-04-23

**Authors:** Rosalia Dambe, John Sande, Doreen Ali, Ben Chilima, Wilfred Dodoli, Charles Michelo, Grace Malenga, Kamija S Phiri

**Affiliations:** Department of Public Health, School of Medicine, University of Zambia, P.O Box 50110, Lusaka, Zambia; National Malaria Control Programme, Ministry of Health, Private Bag 65, Lilongwe, Malawi; National Public Health Reference Laboratory Ministry of Health, Private Bag 65, Lilongwe, Malawi; World Health Organization, P.O. Box 30390, Lilongwe, Malawi; Department of Public Health, College of Medicine, University of Malawi, Private Bag 360, Blantyre, Malawi

**Keywords:** Malawi, Artemether-lumefantrine, Children, Uncomplicated malaria, Efficacy

## Abstract

**Background:**

The resistance of malaria parasites to sulphadoxine-pyrimethamine (SP) in 2007 led to the Malawi Ministry of Health changing to artemether-lumefantrine (AL) as first-line for uncomplicated malaria treatment. This study determined the efficacy and safety of AL for the treatment of uncomplicated *Plasmodium falciparum* malaria among six to 59 months old Malawian children.

**Methods:**

This was a prospective study of children six to 59 months old treated with AL after presenting with uncomplicated malaria in the six health facilities in Malawi. The children were followed up on days 1, 2, 3, 7, 14, 21 and 28 days post-treatment and assessed for clinical and parasitological responses. The Kaplan Meier survival estimate was used to measure the efficacy of AL by calculating the cumulative risk of failure at day 28.

**Results:**

A total of 322 children were recruited into the study across the six sites. The overall intention-to-treat (ITT) polymerase chain reaction (PCR)-corrected cure rate was 93.4%. Per protocol overall PCR-corrected cure rates for the study sites were; Karonga 98.0%, Kawale 97.4%, Machinga 90.2%, Mangochi 95.4% and Rumphi 91.3%. Nkhotakota study site had the lowest cure rate of 78.0%.

**Conclusions:**

There is evidence of good efficacy of AL in Malawi notwithstanding geographical contrasts and this supports the continued use of AL as the first-line treatment for uncomplicated malaria. However there may be need to further investigate the comparatively low efficacy rate found in Nkhotakota district in order to identify possible determinants of treatment failure.

## Background

Malaria is still one of the leading causes of mortality among children under five years of age in sub-Saharan Africa despite efforts to control it [1,2]. Globally deaths due to malaria were estimated at 584 000 with an uncertainty range of 367 000 to 755 000 in 2013 [3]. The prevalence of malaria was 28% among six to 59 months old children in Malawi [4]. Prevention and control of malaria is important in the reduction of malaria burden in Malawi. The Malawi malaria strategic plan for the years 2011 to 2015 aims at moving towards universal coverage of malaria interventions [5]. Malaria is included in the Malawi Millennium Development Goal (MDG) number 6, whose target is to stop and begin to reverse the incidence of malaria and other major diseases by 2015 [6,7].

The World Health Organization (WHO) recommended the use of artemisinin-based combination therapy (ACT) for treatment of uncomplicated malaria [8]. The current first-line fixed dose ACT is presumed to be active against all forms of *Plasmodium* [9,10], including artemether-lumefantrine (AL), artesunate-amodiaquine, artesunate-mefloquine, and artesunate plus sulphadoxine-pyrimethamine (SP). Malawi Ministry of Health changed to AL as first-line drug for uncomplicated malaria treatment based on SP resistance in 2007 [11]. Evidence from other countries show high efficacy and safety of AL [12,13]. However, there has been evidence of resistance to ACT in countries of greater Mekong sub-region [14-16]. Delayed parasite clearance is a sign of artemisinin resistance [17]. Passive surveillance systems are a basis for detection and gathering epidemiological data among countries that are aiming at malaria disease control [18]. Sentinel site monitoring and surveillance of anti-malarial drug efficacy every 24 months assists in containment of resistance of malaria parasite to ACT. The WHO recommends conducting research in resistance to ACT for proper management and understanding of the resistance [14]. The study was conducted to determine AL efficacy and safety for the treatment of uncomplicated *Plasmodium falciparum* malaria among Malawian children aged between six to 59 months.

## Methods

### Study design and setting

This was a prospective study conducted between the months of March to June 2010 aimed at monitoring the efficacy and safety of AL for the treatment of uncomplicated *P. falciparum* malaria in Malawian children. Malawi is in south-eastern Africa. The climate is tropical-continental with some maritime influences and a rainy season varying between November and April. Malaria is endemic in Malawi, with a prevalence of 28% among children under five years of age [4]. The study took place in Malawi at the following study sites: Rumphi, Mangochi, Nkhotakota, Machinga, Lilongwe and Karonga. Children six to 59 months old, presenting with uncomplicated *P. falciparum* malaria at the health centre in the selected study sites were recruited in the study.

### Participant recruitment and follow up

Study participants were screened as they presented to the health facility. The inclusion criteria for the study were: children aged from six to 59 months, presence of mono-infection with *P. falciparum* detected by microscopy with parasite density of 1,000 - 200,000/μl asexual forms, axillary temperature ≥37.5 °C or history of fever for the previous 24 hours, guardians willing to return for weekly routine and unscheduled visits, planning to remain in the study area during the study and provided informed consent. The exclusion criteria for the study were: presence of general danger signs or severe *P. falciparum* malaria in the children as per WHO classification, mixed or mono-infection with other *Plasmodium* species, severe malnutrition (child growth standard below –3 z-score, or symmetrical oedema involving the feet or mid-upper arm circumference <110 mm), other diseases like measles, acute lower respiratory tract infection, severe diarrhoea with dehydration, cardiac, renal and hepatic diseases. Further exclusion included regular medication, which might have interfered with anti-malarial drugs pharmacokinetics and history of contraindications or hypersensitivity reactions to AL. Assuming that treatment failure rate of AL in Malawi will be about 5%. At a confidence level of 95% and a precision around the estimate of 5%, a minimum of 73 patients were to be included. With a design effect of 2.0, a number of 146 participants were reached. Allowing for a 20% loss to follow-up and withdrawals, a minimum 176 participants was to be included in the study.

Children enrolled after meeting the inclusion criteria were given a personal identification number after guardian signed an informed consent. All children were treated with AL. Regular tablets of AL (Coartem®, Novartis) were administered orally according to weight bands using a standard dose of 2mg/kg artemether with 10mg/kg lumefantrine (co-formulated tablets) twice daily on Days 0, 1 and 2 [8]. All six doses were directly observed by a research nurse or clinician. If a patient vomited twice within 30 minutes, he or she would receive 10mg (0.2ml) of quinine per kg body weight as determined by the clinician according to the national treatment guidelines.

The study was conducted for a period of 28 days. Day 0 was designed as the day patients were enrolled and received the first dose of AL. Follow up was from Day 1, 2, 3, 7, 14, 21 and 28. During the follow up visits, children had clinical and laboratory assessments done. Haemoglobin was determined using a Hemocue®. Thick and thin blood smears were prepared for determination of malaria parasitaemia. Smears were double read and if any discrepancies between the two readers, the slide was read by a third person independently. PCR analysis was conducted at the Malawi Liverpool Wellcome Trust laboratories, which provided a PCR-corrected outcome.

Guardians of participants were advised to return on any day during the follow-up period if symptoms recurred or became severe, or any occurrence of adverse events. International Conference on Harmonization (ICH) Tripartite Guideline for 2003 defines an adverse event as “*any untoward medical occurrence in a patient administered a medicinal product and which does not necessarily have to have a causal relationship with this treatment*” [19].

### Outcomes of the study

The primary outcomes for the study were overall treatment success and overall treatment failure. Treatment outcomes were classified according to WHO guidelines as; early treatment failure (ETF), late clinical failure (LCF), late parasitological failure (LPF), loss to follow-up, withdrawal from the study and adequate clinical and parasitological response (ACPR) [20]. The secondary outcomes were gametocyte carriage, parasitaemia and fever.

### Data management and statistical analysis

Data was collected on paper using a Case Report Form (CRF). These were checked on a daily basis before data entry. Then the data was independently double entered into an Excel database specifically designed by the WHO for anti-malarial efficacy studies. Data was then exported and analysed in STATA. Both intention to treat analysis and per protocol analysis methods were used when analysing the data. Intention to treat analysis estimated the effect of the assigned treatment (AL). Per protocol analysis estimated the effect of adhering to the assigned treatment protocol. A description of baseline characteristics of all participants involved in the study was presented. The Kaplan Meier method was used to measure the efficacy of AL by calculating the cumulative risk of failure at day 28. There was comparison among the study sites Rumphi, Mangochi, Nkhotakota, Machinga, Kawale, and Karonga on: the proportion of lost to follow up patients and those patients withdrawn from the study, proportion of ETF, LCF, LPF, ACPR on day 28, success and failure cumulative incidence rates on day 28 with 95% confidence interval and p value of less than 0.05 was considered significant. The differences in efficacy between sites were assessed using Cox proportional hazards model.

### Ethical consideration

This study was approved by the National Health Sciences Research Committee (Protocol Approval number: NHSRC/725) in Malawi.

## Results

### Baseline characteristics

An overall number of 322 participants were recruited into the study. Intention to treat analysis included loss to follow up and withdrawals in the denominator while per protocol analysis excluded participants withdrawn 16.2% (52/322) and lost to follow up 2.5% (8/322) from the study. Table 1 shows the baseline characteristics of the study participants. The female participants were 50.3% (162/322). The overall mean weight was 11.4 kg (SD 2.7 kg). The overall mean height for the participants was 75 cm (SD 22.6 cm). The mean temperature at recruitment on day 0 was 38.5 degrees Celsius (SD 1.2 degrees Celsius). In the study, four participants were recruited above the inclusion criteria parasite density of 200,000 but were included in the analysis as these children (above limit) did not present with any other WHO criteria for severe malaria. Parasite density showed median of 33,080 (inter-quartile range 12,810 – 76,980 μmL).

**Table 1:** **Baseline characteristics of the study participants of efficacy of AL study among Malawian children**

**Characteristic**	**Study population** ^**1**^
Sex	
Female	50.3% (162/322)
Male	49.6% (160/322)
Age (months)^2^	27.6 (14.3)
Weight (kg) ^2^	11.4 (2.7)
Height (cm)^2^	74.9 (22.6)
Temperature °C^3^	38.5 (1.2)
Parasitaemia median (IQR) ^4^	33,080 (12,810 – 76,980)
Gametocyte carriage	2.1% (7/322)

Notes: ^1^n = 322; ^2^Mean (standard deviation); ^3^At enrolment into the study mean (standard deviation); ^4^Median (Inter-quartile range)Per micro mil at enrolment.

### Temperature and parasite clearance

**Figure 1:**
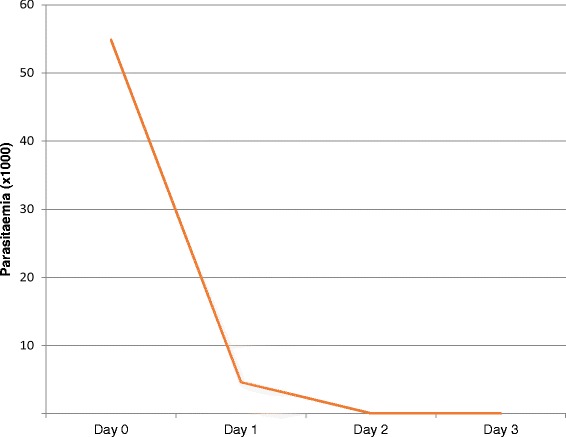
Mean Day 0 to 3 parasitaemia among study participants for all study sites.

All 322 children presented with fever during recruitment into the study with the mean temperature of 38.5°C. All 322 children reported with parasitaemia at recruitment on Day 0. Day 1 reported 68.1% (211/310) children who had not cleared parasites, while Day 2 had 9.1% (28/305) children with parasitaemia. Day 3 had 0.9% (3/305) children reporting with parasitaemia. Figure 1 shows mean Day 0 to 3 parasitaemia for all study sites.

### Efficacy of AL

**Figure 2:**
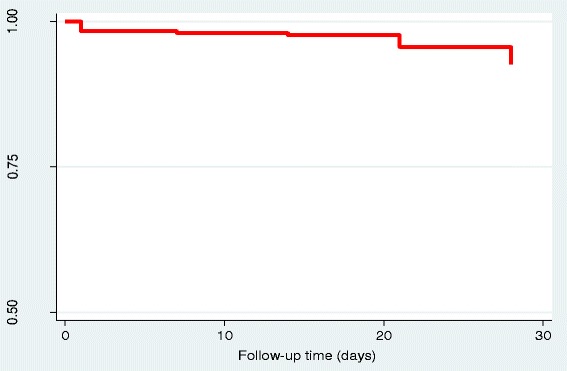
Intention to treat Kaplan-Meier survival estimate of adequate clinical and parasitological response on day 28.

All participants who presented with malaria during the follow-up period had their filter paper sample undergo PCR. The PCR-corrected cure rates were measured. Using intention to treat analysis, ETF was reported among 1.6% (5/320) study participants. LPF was reported among 0.9% (3/320) study participants while LCF was reported among 4.1% (13/320) study participants during the 28 days follow up period. This makes a total of 21 children with treatment failure and total time at risk of 7,926 days. The incident rate (hazard function) was estimated as 2.65 per 1,000 persons per day with the assumption that the incident rate remains constant. Intention-to-treat Kaplan-Meier survival estimate curve is displayed in Figure 2 and the ACPR proportion was 0.934.

### Efficacy of AL by study site

The treatment outcomes by study site were as follows; Nkhotakota study site had the highest LCF of 6, while Machinga study site had 3 LCF and Karonga had 0 LCF. Karonga, Machinga and Nkhotakota study sites showed one child with ETF in each study site while Kawale and Mangochi had no children observed to ETF. There were three children with LPF, one in Mangochi and two in Nkhotakota which were confirmed by PCR (Table 2). Table 3 shows the adjusted hazard ratios; Nkhotakota study site risk of treatment failure was significantly higher as compared to Karonga study site.

**Table 2:** **Treatment outcomes by study site**

	**Study site**					
**Outcome** ^**1**^	**Karonga**	**Kawale**	**Machinga**	**Mangochi**	**Nkhotakota**	**Rumphi**
	**n = 52**	**n = 54**	**n = 55**	**n = 57**	**n = 49**	**n = 53**
ACPR^2^	49	38	37	41	32	42
ETF^3^	1	0	1	0	1	2
LCF^4^	0	1	3	1	6	2
LPF^5^	0	0	0	1	2	0
LFU^6^	1	2	1	2	1	1
WTH^7^	1	13	13	12	7	6

^1^Treatment outcomes; ^2^Adequate Clinical and Parasitological Response; ^3^Early Treatment Failure; ^4^Late Clinical Failure; ^5^Late Parasitological Failure; ^6^Loss to follow-up; ^7^Withdrawals; N = 320, 2 missing.

**Table 3:** **Risk of treatment failure in six study sites adjusted for age and sex**

**Characteristic**	**PCR-corrected cure rate**	**Hazard ratio** ^**1**^ **(95% CI)**	**P value**
**Per protocol analysis**			
Age group(months)			
6 - 12	86.5% (32/37)	1	-
13 - 24	91.1% (82/90)	0.79 (0.25, 2.46)	0.680
25 - 36	94.1% (48/51)	0.46 (0.11, 1.95)	0.289
37 - 59	93.7% (74/79)	0.50 (0.14, 1.76)	0.283
Sex			
Female	90.5% (114/126)	1	
Male	93.3% (125/134)	0.71 (0.30, 1.72)	0.457
Study site			
Karonga	98.0% (49/50)	1	-
Kawale	97.4% (38/39)	1.25 (0.08, 20.28)	0.873
Machinga	90.2% (37/41)	4.76 (0.52, 43.34)	0.166
Mangochi	97.4% (41/43)	2.08 (0.19, 23.16)	0.550
Nkhotakota	78.1% (32/41)	10.60 (1.33, 83.94)	0.025
Rumphi	91.3% (42/46)	4.33 (0.48, 39.03)	0.191

^1^Tested by Cox regression (95% Confidence Interval).

### Gametocyte carriage

Gametocytes were found in peripheral blood of seven children at recruitment into the study. One child each was found with gametocytes on Day 7 and 21. No gametocyte carriage was reported on any other follow-up day.

### Occurrence of adverse events

**Figure 3:**
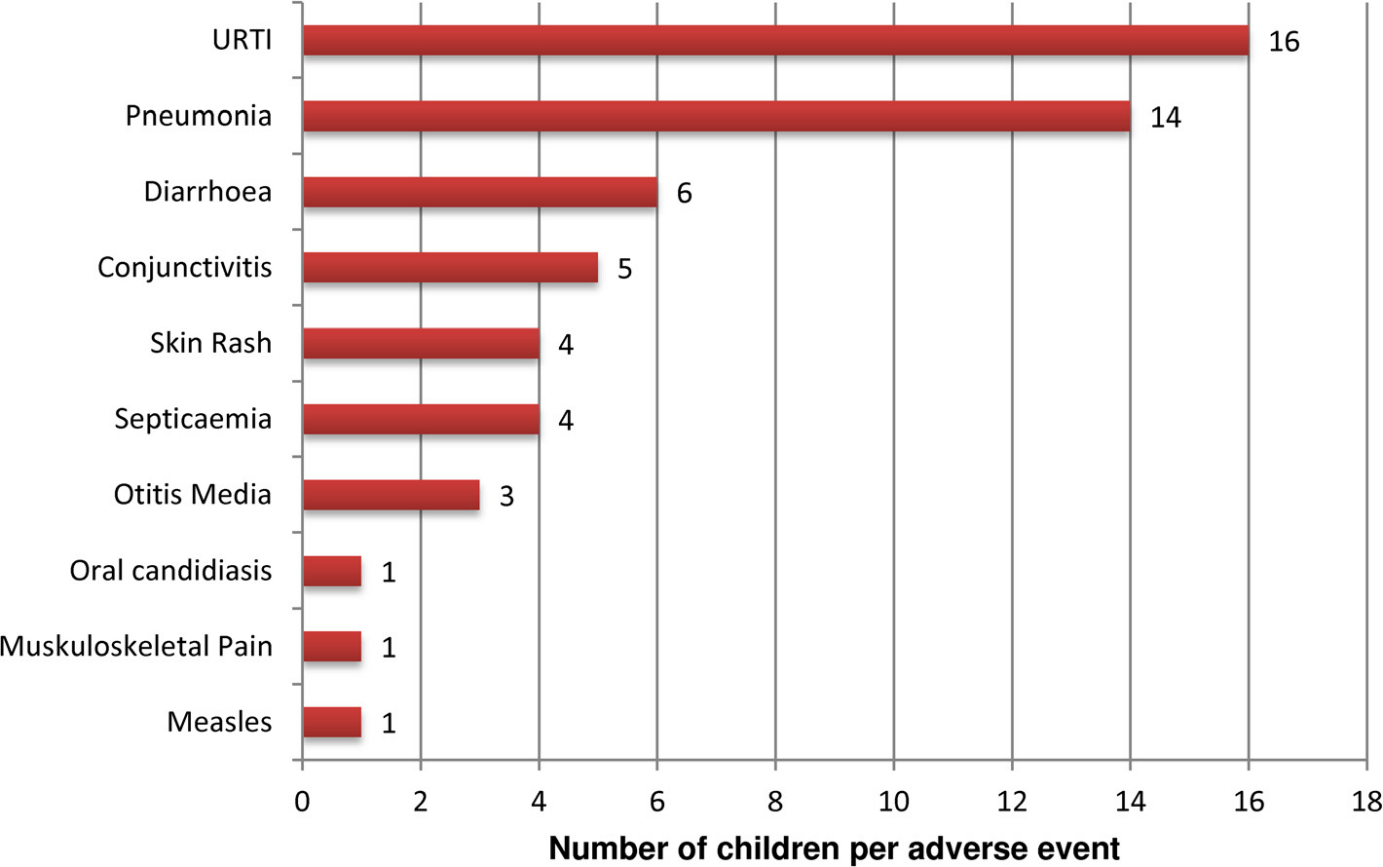
Adverse events among children during the 28 days study period.

A number of adverse events occurred during the 28 day follow-up period however none of the events were related to the effect of AL (Figure 3). The most common occurring adverse events were upper respiratory tract infection (URTI) and pneumonia.

## Discussion

The study showed that the overall efficacy of AL after PCR correction was high in Malawi at 93.4%. This corresponds with other studies done in other countries of Africa on the efficacy of AL [12,13,21,22]. A study done in Ethiopia, although it had a slightly different study design and the follow up period was up to 42 days [21] as compared to the 28 days follow up in this study, had comparable findings. Correction by PCR enables differentiation between recurrence and recrudescence of the initial infection from re-infection. This present study showed that there were a few treatment failures, although most of them were re-infections as opposed to recrudescence. This is of particular concern in areas with very intense malaria transmission where anti-malarial drugs with longer half-life may offer the advantage of preventing re-infection but also be a target for drug resistance development. The very low efficacy rate observed in Nkhotakota is of particular concern. It is clear that this is a site with intense malaria transmission and where other malaria interventions have been carried out. Treatment failure is when malaria parasites fail to clear and malaria clinical symptoms fail to be cured even though anti-malarial drugs have been taken [14]. It can be influenced by several factors more often a decrease in drug concentrations [23]. A limitation of this study was the unavailability of lumefantrine levels on Day 7 which would help further clarify reasons for some of the treatment failures, as there are many other factors that may cause poor absorption of the drugs hence decreasing the bioavailability of AL in the blood. The other limitation was the power of the study; this study was under-powered for site specific analysis.

There was a dramatic reduction in parasitaemia in the study participants after only one day’s dose of AL. This is consistent with other studies that have shown that the artemisinin derivative act more rapidly than other types of anti-malarial, both in killing parasites and in inhibiting their major metabolic processes, such as glycolysis, nucleic acid and protein synthesis [24]. They also attack the broadest age range of parasites, from the smallest rings that have recently invaded erythrocytes to more mature stages of parasites such as developing trophozoites and schizonts [24,25]. Their relatively broad stage-specificity of action extends to an ability to impede the development of gametocytes [26]. This contrasts with some other widely used anti-malarial classes such as the 4-aminoquinolines or anti-folates, which do not have the potential to interrupt transmission of malaria. Furthermore, artemisinin drugs inhibit the ability of maturing parasites to make the red cell surface sticky (cytoadherence to endothelial cells) much more effectively than most other anti-malarial drugs [27]. Adverse events reported during the study period were not related to the effect of AL hence did not show serious drug reactions which might have made AL unsafe to take.

The recommendations for the WHO malaria treatment guidelines in the year 2010 emphasises on switching to another anti-malarial drug when the first-line has more than a 10% overall failure rate [8]. Even though Malawi has not yet reached the 10% mark, resistance to artemisinin was discovered along the Thailand–Myanmar border eight years back [28]. A study conducted among adults and children observed that AL was still efficacious in Southern Laos but increased significant rates of recrudescence were observed among the children [29]. The high failure rate for Nkhotakota study site is of concern. The Ministry of Health should continue monitoring AL efficacy and anti-malarial drug resistance in sentinel surveillance sites.

At policy level, AL should remain to be used as first-line treatment for the treatment of uncomplicated *P. falciparum* malaria in Malawi since the study has demonstrated that AL is safe and has not exceeded the more than 10% cut-off failure rate.

## Conclusion

This study aimed at determining whether AL was efficacious and safe, and investigated the difference in efficacy between the study sites. The findings of the study showed that there was high efficacy of AL in Malawi. Therefore, it is important to monitor efficacy in order to detect and prevent resistance of AL. Surveillance of the established sentinel sites should continue. However there may be need to further investigate the comparatively low efficacy rate found in Nkhotakota district in order to identify possible determinants of treatment failure. Therefore, AL should remain the first-line treatment for uncomplicated malaria among children in Malawi.
